# Biomarkers of Pediatric Cataracts: A Proteomics Analysis of Aqueous Fluid

**DOI:** 10.3390/ijms24109040

**Published:** 2023-05-20

**Authors:** Christos N. Theophanous, Donald J. Wolfgeher, Asim V. Farooq, Sarah Hilkert Rodriguez

**Affiliations:** 1 Department of Ophthalmology and Visual Science, University of Chicago Medical Center, Chicago, IL 60637, USA; 2 Department of Molecular Genetics and Cell Biology, University of Chicago, Chicago, IL 60637, USA

**Keywords:** pediatric cataracts, proteomics, aqueous humor

## Abstract

Cataracts are among the most common causes of childhood vision loss worldwide. This study seeks to identify differentially expressed proteins in the aqueous humor of pediatric cataract patients. Samples of aqueous humor were collected from pediatric and adult cataract patients and subjected to mass spectrometry-based proteomic analysis. Samples of pediatric cataracts were grouped by subtype and compared to adult samples. Differentially expressed proteins in each subtype were identified. Gene ontology analysis was performed using WikiPaths for each cataract subtype. Seven pediatric patients and ten adult patients were included in the study. Of the pediatric samples, all seven (100%) were male, three (43%) had traumatic cataracts, two (29%) had congenital cataracts, and two (29%) had posterior polar cataracts. Of the adult patients, seven (70%) were female and seven (70%) had predominantly nuclear sclerotic cataracts. A total of 128 proteins were upregulated in the pediatric samples, and 127 proteins were upregulated in the adult samples, with 75 proteins shared by both groups. Gene ontology analysis identified inflammatory and oxidative stress pathways as upregulated in pediatric cataracts. Inflammatory and oxidative stress mechanisms may be involved in pediatric cataract formation and warrant further investigation.

## 1. Introduction

Cataracts are among the most common causes of childhood vision loss worldwide [1,2]. The etiologies of pediatric cataracts include infectious causes, metabolic conditions, genetic syndromes, trauma, developmental anomalies, and sporadic development [3]. Cataracts that do not affect vision can be observed, but those that affect vision generally require surgical extraction, which is performed more urgently when they present a higher risk of amblyopia [3]. While the underlying etiologies of pediatric cataracts have been widely described, the biochemical changes associated with pediatric cataract formation are not yet fully understood. Proteomic analysis may allow for the identification of proteins that may be altered in pediatric cataracts, thereby improving our understanding of the underlying pathophysiology of the condition [4].

Previous studies conducted in adult patients have demonstrated that proteomic analysis of aqueous humor can enhance our understanding of eye conditions. For example, proteomic studies conducted on aqueous humor from adult cataracts have demonstrated that proteins are differentially expressed in pseudoexfoliation syndrome compared to controls [5]. Another adult proteomic study revealed specific proteins that were increased in the aqueous humor of glaucoma patients compared to cataract patients, which the authors hypothesized were involved in damage to the trabecular meshwork that leads to glaucoma [6].

In the pediatric population, an aqueous humor analysis from infants with primary congenital glaucoma found an increase in proteins that play a role in retinoic acid binding and transport, suggesting that factors related to retinoic acid may affect anterior segment development [7]. Another study analyzing vitreous humor from retinopathy-of-prematurity (ROP) patients compared to congenital cataract patients found reduced levels of several proteins including pigment epithelium derived factor (PEDF) and transthyretin, in ROP patients, which may affect angiogenesis in this demographic [8]. Both of these studies offer evidence that evaluating the protein composition of ocular fluids can offer insights into the pathophysiology of pediatric ocular diseases.

Few prior studies have explored the proteomics of aqueous humor in pediatric cataracts. The purpose of this study is to evaluate the hypothesis that certain proteins may be differentially expressed in the aqueous humor of different types of pediatric cataracts.

## 2. Results

The demographics and types of cataracts of the patients included in this study are summarized in Table 1. Notably, all seven pediatric patients were male. Three (43%) had traumatic cataracts, two (29%) had congenital cataracts, and two (29%) had posterior polar cataracts. Five (71%) were unilateral. Of the adult patients, seven (70%) were female. Seven (70%) had predominantly nuclear sclerotic cataracts. Nine (90%) had hypertension, six (60%) had diabetes, and four (40%) had glaucoma or ocular hypertension requiring the use of antihypertensive topical medications.

**Figure 1:**
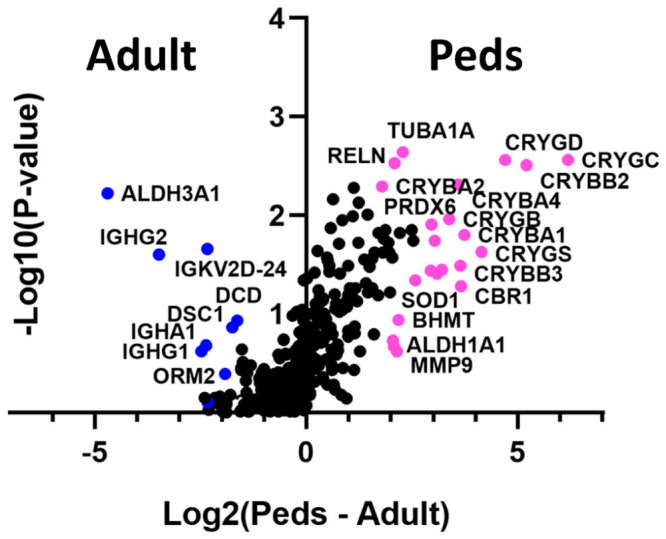
Volcano plot representing proteins that are differentially upregulated in adult versus pediatric samples. The *x*-axis (Log2) represents the fold-change of protein expression, while the *y*-axis (−Log10) represents the *p*-value. Blue dots represent significant hits in the adult population while pink dots represent significant hits in the pediatric population.

Across all samples, 330 proteins were identified. 128 proteins were upregulated in the pediatrics samples, 127 proteins were upregulated in the adult samples, and 75 proteins were shared by both groups. A volcano plot of these protein results is given in Figure 1, and Table 2 and Table 3 list the proteins upregulated in the pediatric and adult samples, respectively, with Log2 values greater than or equal to 1.50.

**Figure 2:**
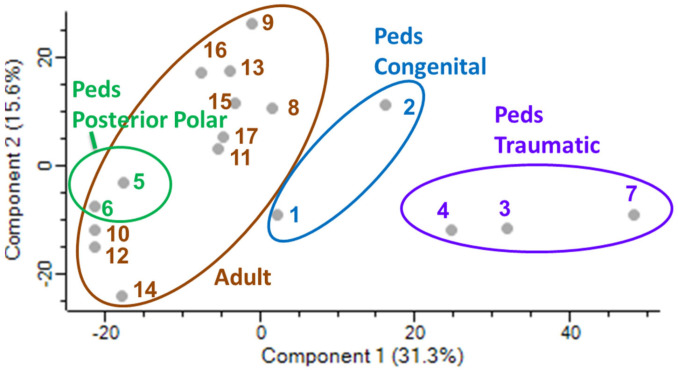
Principal component analysis plot demonstrating clustering according to subtype of pediatric cataract and adult cataract samples.

**Figure 3:**
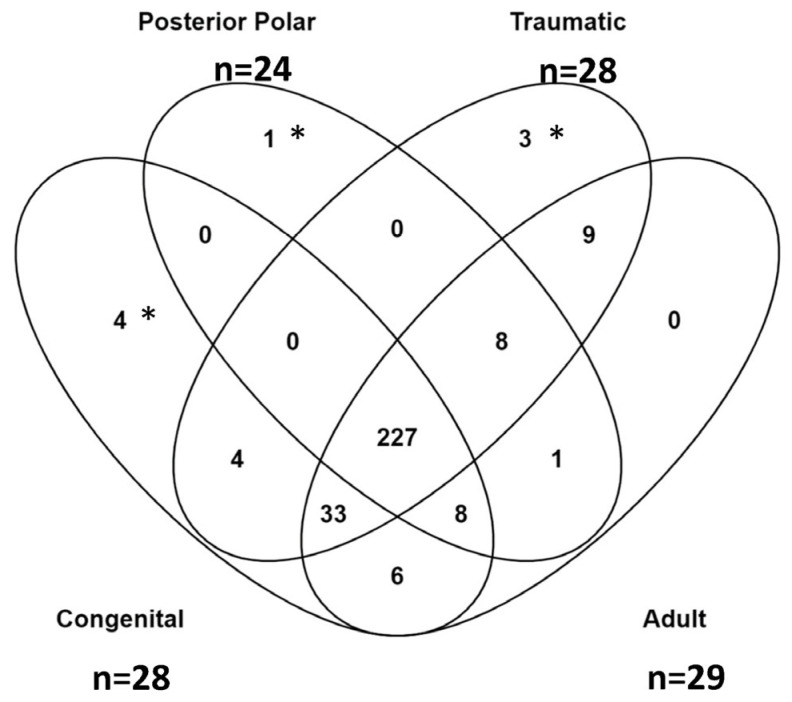
Venn Diagram comparing upregulated proteins between pediatric subtypes and adult samples. Asterisks (*, **, ***) correspond to proteins listed in Table 5.

Differentially expressed proteins were identified according to the subtype of pediatric cataract, including traumatic, congenital, and polar. Figure 2 shows a principal component analysis of each aqueous sample and demonstrates the clustering of the pediatric subtypes compared to the adult samples. Further details of the upregulated proteins in each subtype are shown in Table 4a–c. These tables highlight upregulated proteins in each subgroup with a Log2 value greater than 2.5. The total proteins identified in each subtype are further summarized in Figure 3, which shows the overlap of upregulated proteins arranged by subgroup. The unique upregulated proteins in each subgroup are shown in Table 5. In total, 227 upregulated proteins were shared between all three pediatric subtypes and adult samples. Three proteins were uniquely upregulated in the traumatic cataract samples, four were uniquely upregulated in the congenital cataract samples, and one was uniquely upregulated in the posterior polar cataract samples.

**Figure 4:**
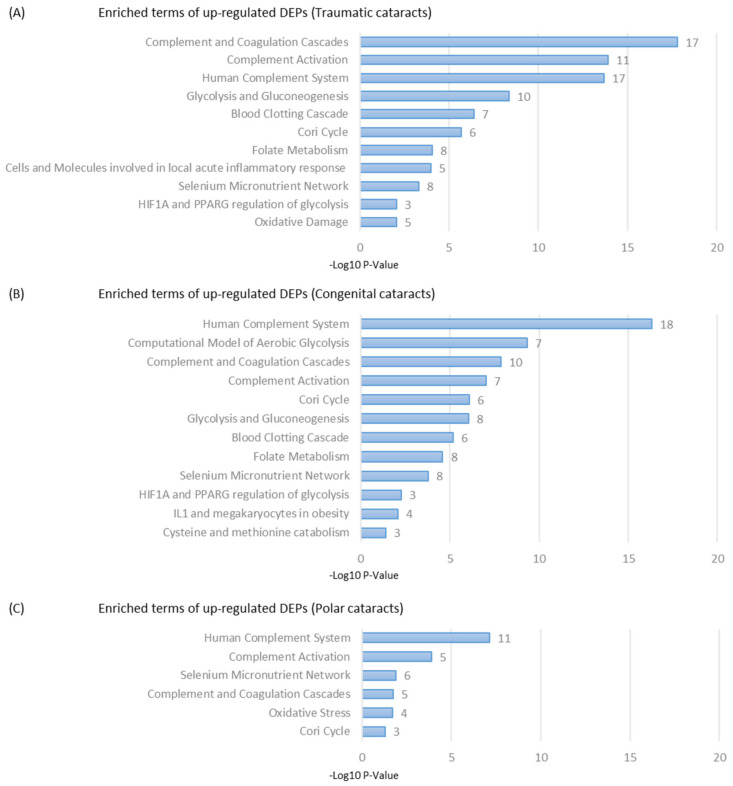
Clusters with enriched terms of upregulated differentially expressed proteins (DEPs) for (**A**) traumatic cataracts, (**B**) congenital cataracts, and (**C**) polar cataracts based on the Wikipaths algorithm. The *x*-axis represents *p* value after negative log10 processing. Numbers next to the bars represent the number of proteins enriched in each cluster.

Gene ontology analysis was performed on each subtype of pediatric cataract samples and was compared to adult controls using the Wikipaths algorithm. The results are summarized in Figure 4. Clusters with the highest −log10 *p* values are ranked for each subtype, and the number of enriched proteins in each cluster are shown.

## 3. Discussion

Pediatric cataracts are an important cause of vision loss worldwide and can impair early visual and neurologic development [2]. A broad list of etiologies for pediatric cataracts has been described in the literature. Between 10% and 29% of pediatric cataracts are attributed to genetic causes [9,10]. Over 200 syndromes have also been associated with the development of pediatric cataracts [11]. To our knowledge, this study is the first to provide an aqueous humor proteomic analysis in this patient population.

In our study, crystallins were the most commonly identified upregulated proteins in the pediatric samples at large. Crystallins comprise an important building block of the human crystalline lens that provides it with clarity and refractive properties [12]. These proteins are subcategorized as α-, β-, and γ-crystallins, each with additional subunits. In our samples, crystallins were most highly upregulated in traumatic cataracts, which we believe is due to the release of crystallins following trauma to the lens capsule. Notably, two of the three traumatic cataract patients in our sample presented with capsular rupture, which likely explains the upregulation of crystallins in those samples. The aqueous samples from patients with congenital cataracts also demonstrated an upregulation of crystallins. Prior genetic studies have demonstrated that crystallin genes are the most frequently identified mutations in patients with isolated congenital cataracts [11]. Mutations in the crystalline genes are thought to result in rapid protein aggregation and opacification of the lens [13]. Crystallins were not significantly upregulated among the samples from patients with posterior polar cataracts. These cataracts predominantly involve focal areas of the posterior capsule rather than diffuse changes occurring throughout the lens, which may explain this difference.

Gene ontology analysis demonstrated an increased activation of complement pathways among all three subgroups compared to the adult samples. These findings suggest that an inflammatory response may be a component of the pathophysiology of pediatric cataracts. However, the makeup of upregulated inflammatory proteins differed by subtype. In the congenital cataract group, several neutrophils associated with proteins such as neutrophil defensin 2 and 3, neutrophil elastase, and neutrophil gelatinase-associated lipocalin were upregulated. Other acute-phase reactants including C-reactive protein were also upregulated in these samples. These findings suggest there may be an acute inflammatory component of the progression of these cataracts.

Congenital cataract samples also demonstrated an upregulation of C4 proteins, while posterior polar cataracts presented upregulated C3 proteins and posterior polar C1r proteins. Of note, adult cataract samples showed an upregulation of C9 protein, indicating some complement activation as well. These differences may suggest variation in the inflammatory cascades involved in each cataract subtype.

Pathway enrichment analysis also indicated involvement of the selenium micronutrient network in all three subtypes of pediatric cataracts. Selenium is a micronutrient with a previously established role in ocular health [14,15]. Prior studies in mice have shown that selenium deficiency through target gene disruption of glutathione peroxidase (G-Px) was associated with cataract formation [14]. Glutathione peroxidase, a selenoprotein, is involved in reducing oxidative stress and protects against nitrogen species [16]. The role of the selenium micronutrient pathway in cataract formation remains poorly understood but may be more activated in pediatric cataract cases.

Alongside the role of glutathione peroxidase, traumatic and polar cataracts also demonstrated an activation of oxidative stress pathways. The association between oxidative stress and cataract formation has been documented in the literature. Prior studies have identified oxidation-induced protein modifications, lipid peroxidation, and decreased levels of protective enzymes such as superoxide dismutase, glutathione reductase, and glutathione peroxidase in cataract formation [17,18]. Notably, in our traumatic samples, both glutathione peroxidase and superoxide dismutase were upregulated. The specific role of oxidative damage in the progression of pediatric cataracts is still unclear, but these pathways may warrant further investigation.

Our study has several significant limitations. Primarily, our control group does not include aqueous samples isolated from healthy pediatric patients due to the ethical limitations of performing anterior chamber paracentesis on otherwise healthy patients. While we recognize that adult aqueous samples of patients undergoing cataract surgery are not a perfect comparison group, we believe they provide the most reasonable comparison possible given this ethical limitation. Secondly, the generalizability of the proteomic results is limited by the number of pediatric samples collected. It is unclear how many of the differences between subtypes of pediatric cataracts are due to cataract pathophysiology or due to age, gender, or other underlying medical conditions of the samples collected. We believe the clustering of protein makeup via our classification lends credibility to the analysis using our chosen subtypes, but a larger sampling study would be needed to confirm these findings. Additionally, as previously discussed, the range of etiologies for pediatric cataracts is broad, and our study is unable to identify differences between these broad types of cataracts. Further studies are needed to investigate the role of particular pathways and proteins in the pathogenesis of pediatric cataracts.

## 4. Materials and Methods

### 4.1. Approval

The study received approval from the University of Chicago Institutional Review Board (IRB) and was conducted in accordance with the ethical standards of the Declaration of Helsinki. All participants or legal guardians signed informed consent and did not receive any stipends. All participants were assured that refusal to participate would not impact their care.

### 4.2. Patients and Sampling

A total of 7 pediatric patients and 10 adult patients with cataracts were enrolled in the study. Inclusion criteria included patients with visually significant cataracts for whom surgery was recommended. For adult patients, surgical candidacy was based on reduction in best-corrected visual acuity to worse than 20/40 or significant functional impairment attributable to cataract progression. For patients with bilateral cataracts, only 1 eye was included in the analysis. Patients with prior glaucoma filtering or shunt surgeries were excluded from the study as it was thought these procedures could affect aqueous fluid flow and protein profiles. Of note, aqueous samples from adult cataract surgery patients were used as the comparison group for pediatric cataract patients because of the ethical concerns of performing anterior chamber paracentesis on healthy adults or children, as this procedure carries risk of endophthalmitis, hypotony, and mechanical injury to intraocular structures. The use of adult cataract surgery patient samples to categorize standard protein makeup of aqueous humor is consistent with previously published studies [19].

Samples were collected during the initial portion of cataract surgery. After a paracentesis wound was made, a small amount of human aqueous humor (hAH), typically between 30–100 µL, was aspirated with a sterile syringe and 30-gauge cannula. The syringe was stored at −80 °C until processing.

### 4.3. Sample Preparation and SDS-Gel Purification

Protein concentration of hAH samples was determined via Bradford Protein Assay, and ~20 µg of sample (7 to 10 µL) was loaded onto a 12% MOPS buffered SDS-PAGE gel (Invitrogen, Waltham, MA, USA) and run for 6 min at 200 V, resulting in a ~2 cm “gel plug”. The gel was stained with Pierce Imperial Stain (Thermo Fisher Scientific, Waltham, MA, USA) for 1 h at room temperature and de-stained overnight in dH_2_O at 4 °C. The gel plugs for each sample to be analyzed were excised as follows.

### 4.4. In-Gel Trypsinization and LC-MS/MS Sample Preparation

Gel Samples were excised by sterile razor blade and chopped into ~1 cubic millimeter pieces. Each section was washed in distilled H_2_O and de-stained using 100 mM of NH_4_HCO_3_ at pH7.5 in 50% acetonitrile. A reduction step was performed via addition of 100 μL of 50 mM NH_4_HCO_3_ (pH 7.5) and 10 μL of 200 mM tris (2-carboxyethyl) phosphine HCl at 37 °C for 30 min. The proteins were alkylated via the addition of 100 μL of 50 mM iodoacetamide that was prepared fresh in 50 mM NH_4_HCO_3_ (pH 7.5) buffer and allowed to react in the dark at 20 °C for 30 min. Gel sections were washed in water and then acetonitrile; then, they were vacuum-dried. Trypsin digestion was carried out overnight at 37 °C with 1:50–1:100 enzyme/protein ratio of sequencing-grade-modified trypsin (Promega, Madison, WI, USA) in 50 mM NH_4_HCO_3_ (pH 7.5) and 20 mM CaCl_2_. Peptides were extracted first with 5% formic acid and then with 75% ACN:5% formic acid; then, they were combined and vacuum dried. Digested peptides were cleaned on a C_18_ column (Pierce), speed-vacuumed, and sent for LC-MS/MS to the Proteomics Core at Mayo Clinic. High-performance liquid chromatography (HPLC) was then performed following previously published protocols [20].

### 4.5. LC-MS/MS Data Acquisition and Analysis

The samples were analyzed via data-dependent electrospray tandem mass spectrometry (LC-MS/MS) on a Thermo Q-Exactive Orbitrap mass spectrometer with parameters consistent with previously published methods [21].

All LC-MS/MS *. raw data files were analyzed with MaxQuant version 1.5.2.8, searching against the SPROT Human database (Download 5/1/2020 with isoforms) *. fasta sequence using the following criteria: Label-Free Quantification (LFQ) was selected for Quantitation with a minimum of 1 high-confidence peptide to assign LFQ Intensities. Trypsin was selected as the protease, with maximum missing cleavage set to 2. Carbamiodomethyl (C) was selected as a fixed modification. Variable modifications were set to Oxidization (M), Formylation (N-term), and Deamidation (NQ). Orbitrap mass spectrometer was employed using an MS error of 20 parts per million and an MS/MS error of 0.5 Da. A 1% False Discovery Rate (FDR) cutoff was selected for peptide, protein, and site identifications. Ratios were reported based on the LFQ Intensities of protein peak areas determined by MaxQuant (version 1.5.2.8) and reported in proteinGroups.txt. The proteingroups.txt file was processed in Perseus (version 1.6.7). Proteins were removed from this results file if they were flagged by MaxQuant as “Contaminants”, “Reverse”, or “Only identified by site”. LFQ peak intensities were Log2-transformed and median-normalized, and missing values were imputed via default settings in Perseus. The data were then grouped via patient age into Peds or Adult, Log2 ratio was determined (Peds/Adult), and significance was determined at 20% up or down. The number of significant hits for Peds was ≥0.26, the number of significant hits for Adult was ≤−0.32, and any remaining hits were grouped into a common bin. Systems biology analysis was performed in DAVID.

The full proteomic data set was uploaded to the ProteomeXchange repository (https://www.ebi.ac.uk/pride/archive/PXD022529, accessed on 1 May 2020).

### 4.6. Statistical Analysis

Results were searched on MaxQuant against Human Uniprot database at 20 parts per million mass error, which was filtered at 1% FDR cutoff (i.e., 99% real hits). Pediatric and adult samples were grouped, and Log2 of the LFQ intensity (protein abundance) was calculated with a comparison run between pediatric and adult samples. This analysis was also repeated with pediatric samples grouped by type of cataract.

## 5. Conclusions

Our study is among the first to describe proteomic differences in aqueous humor between pediatric and adult cataracts. Gene ontology analysis revealed that inflammatory and oxidative stress pathways were upregulated in pediatric cataracts. The pathways upregulated in these cataracts may provide insight for future studies seeking to better understand the underlying mechanisms of pediatric cataract formation and targets for potential future therapeutics.

## Figures and Tables

**Table 1:** Patient demographics.

Case	Age (yr)	Gender	Cataract Type	Laterality	Additional Features
Pediatric					
1	0.7	M	Congenital	Bilateral	Developmental delay; positive Rubella titers
2	2	M	Congenital	Bilateral	Developmental delay
3	5	M	Traumatic	Unilateral	History of ruptured globe with violation of lens capsule
4	5	M	Traumatic	Unilateral	History of elastic injury to the eye but no lens capsule violation
5	9	M	Posterior Polar	Unilateral	Visually significant cataract; fellow eye with Mittendorf dot
6	9	M	Posterior Polar	Unilateral	Dense visually significant cataract; lens clear in fellow eye
7	10	M	Traumatic	Unilateral	History of elastic injury to eye with associated lens capsule violation
Adult					
8	55	F	PSC	Bilateral	HTN; DM; PDR; VH; Multiple intravitreal Avastin injections and PRP
9	58	M	Cortical	Bilateral	HTN; DM; BRVO in fellow eye
10	60	F	NS	Bilateral	HTN; Pars planitis and VH requiring PPV in fellow eye
11	61	F	NS	Bilateral	HTN; ESRD; Ocular HTN on single antihypertensive medication
12	68	F	NS	Bilateral	HTN; CKD
13	73	M	NS	Bilateral	HTN; DM; POAG on single antihypertensive medication
14	74	F	NS	Bilateral	DM; Narrow angle glaucoma on single antihypertensive medication
15	75	M	Cortical	Bilateral	HTN; DM
16	79	F	NS	Bilateral	HTN; DM
17	87	F	NS	Bilateral	HTN; POAG on single antihypertensive medication

HTN—Hypertension; DM—Diabetes Mellitus; PDR—Proliferative Diabetic Retinopathy; VH—Vitreous Hemorrhage; PRP—Panretinal photocoagulation; BRVO—Branch Retinal Vein Occlusion; PPV—Pars Plana Vitrectomy; ESRD—End-stage Renal Disease; CKD—Chronic Kidney Disease; POAG—Primary Open Angle Glaucoma; NS—Nuclear Sclerosis; PSC—Posterior subcapsular cataract.

**Table 2:** List of upregulated proteins in pediatric samples compared to adult samples.

Gene Name	Protein Names	Log2 (Fold Change)	−Log10 (*p* Value)	Mol. Weight [kDa]
CRYGC	Gamma-crystallin C	6.19	2.56	20.9
CRYBB2	Beta-crystallin B2	5.21	2.51	23.4
CRYGD	Gamma-crystallin D	4.70	2.56	20.7
CRYGS	Beta-crystallin S	4.14	1.63	21.0
CRYBA1	Beta-crystallin A3	3.74	1.80	25.2
CBR1	Carbonyl reductase [NADPH] 1	3.66	1.28	30.4
CRYBB3	Beta-crystallin B3	3.64	1.49	24.3
CRYBA4	Beta-crystallin A4	3.59	2.31	22.4
CRYGB	Gamma-crystallin B	3.38	1.96	20.9
CRYAB	Alpha-crystallin B chain	3.21	1.45	20.2
CRYBB1	Beta-crystallin B1	3.09	1.41	28.0
CRYAA	Alpha-crystallin A chain	3.04	1.74	19.9
PRDX6	Peroxiredoxin-6	2.96	1.91	25.0
GSS	Glutathione synthetase	2.94	1.44	52.4
SOD1	Superoxide dismutase [Cu-Zn]	2.58	1.34	15.9
PARK7	Protein DJ-1	2.53	1.74	19.9
PEBP1	Phosphatidylethanolamine-binding protein 1; Hippocampal cholinergic neurostimulating peptide	2.49	1.85	21.1
TUBA1A	Tubulin alpha-1A, -1B, -1C, -3E chain	2.28	2.64	46.3
SERPINB6	Serpin B6	2.21	1.82	42.6
BHMT	Betaine--homocysteine S-methyltransferase 1	2.18	0.94	45.0
MMP9	Matrix metalloproteinase-9	2.14	0.62	78.5
RELN	Reelin	2.09	2.53	388.4
IGKC	Ig kappa chain C region	2.07	0.67	11.8
ALDH1A1	Retinal dehydrogenase 1	2.05	0.73	54.9
TIMP1	Metalloproteinase inhibitor 1	2.03	1.57	16.1
C1QB	Complement C1q subcomponent subunit B	1.98	1.22	24.0
LDHA	L-lactate dehydrogenase A chain	1.96	1.62	36.7
PGK1	Phosphoglycerate kinase 1	1.94	1.72	41.4
CRYGA	Gamma-crystallin A	1.87	1.85	20.9
LGSN	Lengsin	1.83	1.76	21.9
HIST1H4A	Histone H4	1.81	1.62	11.4
CRYBA2	Beta-crystallin A2	1.79	2.29	22.1
BPGM	Bisphosphoglycerate mutase	1.71	1.82	30.0
ALDOA	Fructose-bisphosphate aldolase A	1.70	1.19	39.4
LDHB	L-lactate dehydrogenase B chain	1.61	1.62	37.3
IGHV5-10-1	Immunoglobulin heavy variable 5-10-1	1.60	0.79	12.8
FN1	Fibronectin; Anastellin; Ugl-Y1; Ugl-Y2; Ugl-Y3	1.56	1.56	243.3
SPTAN1	Spectrin alpha chain, non-erythrocytic 1	1.56	1.26	282.8
IGKV3-15	Ig kappa chain V-III region POM	1.50	1.48	12.5

**Table 3:** List of upregulated proteins in adult samples versus pediatric samples.

Gene Name	Protein Names	Log2 (Fold Change)	−Log10 (*p* Value)	Mol. Weight [kDa]
ALDH3A1	Aldehyde dehydrogenase, dimeric NADP-preferring	−4.69	2.22	41.6
IGHG2	Ig gamma-2 chain C region	−3.48	1.60	43.8
IGHG1	Ig gamma-1 chain C region	−2.48	0.62	43.9
LRG1	Leucine-rich alpha-2-glycoprotein	−2.40	0.15	38.2
IGHA1	Ig alpha-1 chain C region	−2.37	0.68	42.8
IGKV2D-24	Immunoglobulin kappa variable 2-24	−2.34	1.66	13.1
F2	Prothrombin; Activation peptide fragment 1; Activation peptide fragment 2	−2.33	0.10	70.0
AMBP	Protein AMBP; Alpha-1-microglobulin; Inter-alpha-trypsin inhibitor light chain; Trypstatin	−2.31	0.03	39.0
C9	Complement component C9, C9a, C9b	−2.16	0.15	63.2
RBP4	Retinol-binding protein 4	−2.05	0.08	23.0
HBB	Hemoglobin subunit beta; LVV-hemorphin-7; Spinorphin	−2.04	0.16	16.0
HPX	Hemopexin	−1.98	0.02	51.7
ORM1	Alpha-1-acid glycoprotein 1	−1.98	0.01	23.5
HBA1	Hemoglobin subunit alpha	−1.94	0.02	15.3
ORM2	Alpha-1-acid glycoprotein 2	−1.92	0.39	23.6
SPARCL1	SPARC-like protein 1	−1.89	0.08	61.8
PLG	Plasminogen; Plasmin heavy chain A; Activation peptide; Angiostatin; Plasmin light chain B	−1.89	0.00	90.6
IGHM	Ig mu chain C region	−1.79	0.18	49.4
DSC1	Desmocollin-1	−1.75	0.86	93.8
DCD	Dermcidin; Survival-promoting peptide; DCD-1	−1.63	0.93	11.3
ITIH4	Inter-alpha-trypsin inhibitor heavy chain H4	−1.56	0.01	103.4
TNS1	Tensin-1	−1.54	0.45	185.7
APP	Amyloid beta A4 protein; Beta-amyloid protein 42, 40; C83; C80; Gamma-secretase C-terminal fragment 59, 57, 50; C31	−1.54	0.43	75.1
ANXA2	Annexin; Annexin A2; Putative annexin A2-like protein	−1.52	0.64	16.5
DSG1	Desmoglein-1	−1.50	0.51	113.8

**Table 4:** (**a**) Upregulated proteins in congenital cataracts. (**b**) Upregulated proteins in posterior polar cataracts. (**c**) Upregulated proteins in traumatic cataracts.

(a)		
Gene Names	Protein Names	Log2
CRYGC	Gamma-crystallin C	7.56
CRYBB1	Beta-crystallin B1	7.40
CRYGS	Beta-crystallin S	5.34
MMP9	Matrix metalloproteinase-9	4.93
MPO	Myeloperoxidase	4.43
DEFA3	Neutrophil defensin, 2, 3	4.35
HIST1H4A	Histone H4	4.25
ELANE	Neutrophil elastase	3.88
LTF	Lactotransferrin	3.79
C4BPA	C4b-binding protein alpha chain	3.79
LCN2	Neutrophil gelatinase-associated lipocalin	3.45
HIST1H2BN	Histone H2B	3.44
IGHV3-43D	Ig heavy chain V-III region DOB	3.28
COL1A2	Collagen alpha-2(I) chain	3.27
KLKB1	Plasma kallikrein	3.27
CRYBA1	Beta-crystallin A3	3.26
IGLV1-40	Ig lambda chain V-I region NEWM	3.18
CRP	C-reactive protein	3.11
CRYGD	Gamma-crystallin D	3.06
SPARC	SPARC	2.91
TPI1	Triosephosphate isomerase	2.88
LDHB	L-lactate dehydrogenase	2.84
HP	Haptoglobin	2.83
IGLV3-10	Immunoglobulin lambda variable 3-10	2.77
IGLV1-47	Ig lambda chain V-I	2.66
IGHV6-1	Immunoglobulin heavy variable 6-1	2.64
LCP1	Plastin-2	2.62
**(b)**		
**Gene Names**	**Protein Names**	**Log2**
ABI3BP	Target of Nesh-SH3	4.64
COL9A2	Collagen alpha-2(IX) chain	4.62
XP32	Skin-specific protein 32	3.82
LTBP2	Latent-transforming growth factor beta-binding protein 2	3.54
PKP1	Plakophilin-1	3.26
CDSN	Corneodesmosin	3.25
COL1A2	Collagen alpha-2(I) chain	3.23
DSP	Desmoplakin	3.14
SERPINB12	Serpin B12	3.14
DSG1	Desmoglein-1	3.13
SERPINB3	Serpin B3; Serpin B4	3.01
IGKV1-13	Immunoglobulin kappa variable 1-13	2.99
ENPP2	Ectonucleotide pyrophosphatase/phosphodiesterase family member 2	2.96
HIST1H2BN	Histone H2B	2.96
CRTAC1	Cartilage acidic protein 1	2.96
PRDX1	Peroxiredoxin-1	2.95
JUP	Junction plakoglobin	2.92
CPAMD8	C3 and PZP-like alpha-2-macroglobulin domain-containing protein 8	2.90
SPOCK2	Testican-2	2.86
KPRP	Keratinocyte proline-rich protein	2.74
FABP5	Fatty-acid-binding protein, epidermal	2.71
TGM3	Protein-glutamine gamma-glutamyltransferase E	2.64
SPON1	Spondin-1	2.63
**(c)**		
**Gene Names**	**Protein Names**	**Log2**
CRYGC	Gamma-crystallin C	11.08
CRYBB1	Beta-crystallin B1	10.34
CRYBA1	Beta-crystallin A3	9.17
CRYGS	Beta-crystallin S	8.38
PARK7	Protein DJ-1	8.21
CRYGD	Gamma-crystallin D	8.16
CRYAB	Alpha-crystallin B chain	7.97
CBR1	Carbonyl reductase (NADPH) 1	7.82
CRYGB	Gamma-crystallin B	7.50
CRYBB2	Beta-crystallin B2	7.20
CRYBA4	Beta-crystallin A4	6.81
PRDX6	Peroxiredoxin-6	6.69
GSS	Glutathione synthetase	6.02
SERPINB6	Serpin B6	5.99
CRYAA	Alpha-crystallin A	5.97
CRYBB3	Beta-crystallin B3	5.79
SOD1	Superoxide dismutase (Cu-Zn).	5.66
PGK1	Phosphoglycerate kinase 1	5.63
ALDH1A1	Retinal dehydrogenase 1	5.56
CRYGA	Gamma-crystallin A	4.96
FN1	Fibronectin; Anastellin; Ugl-Y1; Ugl-Y2; Ugl-Y3	4.87
BPGM	Bisphosphoglycerate mutase	4.44
BHMT	Betaine--homocysteine S-methyltransferase 1	4.18
SORD	Sorbitol dehydrogenase	4.12
PEBP1	Phosphatidylethanolamine-binding protein 1; Hippocampal cholinergic neurostimulating peptide	4.04
LGSN	Lengsin	3.95
TF	Serotransferrin	3.92
C1R	Complement C1r	3.83
PGAM1	Phosphoglycerate mutase 1, 2, 4	3.75
GDI2	Rab GDP dissociation inhibitor beta	3.44
HSPB1	Heat shock protein beta-1	3.25
IGKV2D-28	Ig kappa chain V-II region FR; Ig kappa chain V-II region Cum	3.25
ITIH2	Inter-alpha-trypsin inhibitor heavy chain H2	3.20
ALDOA	Fructose-bisphosphate aldolase A	3.01
PON1	Serum paraoxonase/arylesterase 1	2.95
FGA	Fibrinogen alpha chain; Fibrinopeptide A	2.91
APOA2	Apolipoprotein A-II	2.91
FABP5	Fatty-acid-binding protein, epidermal	2.63
LTBP2	Latent-transforming growth factor beta-binding protein 2	2.57

**Table 5:** Unique upregulated proteins in congenital, posterior polar, and traumatic cataract samples. Asterisks (*, **, ***) correspond to protein categories in Figure 3.

Gene Names	Protein Names	Log2
Congenital *		
ARHGDIB	Rho GDP-dissociation inhibitor 2	2.37
PFN1	Profilin-1	1.84
IGLV2-14	Ig lambda chain V-II region TOG	1.53
ADIPOQ	Adiponectin	1.47
Posterior Polar **		
NDRG4	Protein NDRG4	−0.27
Traumatic ***		
CBR1	Carbonyl reductase (NADPH) 1	7.82
LGSN	Lengsin	3.95
BASP1	Brain acid-soluble protein 1	0.82

## Data Availability

Due to the nature of this research, the participants of this study did not agree to have their data shared publicly, so supporting data are not available.
